# The Growing Trend of Moderate Preterm Births: An Ecological Study in One Region of Brazil

**DOI:** 10.1371/journal.pone.0141852

**Published:** 2015-11-03

**Authors:** Rosana Rosseto de Oliveira, Emiliana Cristina Melo, Larissa Pereira Falavina, Thais Aidar de Freitas Mathias

**Affiliations:** 1 Department of Nursing, Graduate Nursing Program, State University of Maringá, Paraná, Brazil; 2 Department of Nursing, Northern Paraná State University, Bandeirantes, Paraná, Brazil; 3 School of Nursing, Nursing program, State University of Maringá, Paraná, Brazil; 4 Department of Nursing, Public Health, State University of Maringá, Paraná, Brazil; Hôpital Robert Debré, FRANCE

## Abstract

**Background:**

Preterm birth is a serious public health problem, as it is linked to high rates of neonatal and child morbidity and mortality, with Brazil listed among the countries with the ten highest numbers of premature births. Nonetheless, knowledge is scarce regarding prematurity and associated factors in mid-sized cities. The objective of this study was to analyze the trend of preterm births and associated factors in a municipality located in the state of Paraná, Brazil.

**Methods:**

This was an ecological time series study of births recorded into the Live Birth Information System for residents of Maringá, Paraná, Brazil, between 2000 and 2013. The polynomial regression model was used for trend analysis of preterm birth, characteristics of the mother, gestation and delivery, and newborn. The association with preterm birth was analyzed using odds ratio (OR).

**Results:**

A total of 61,634 live births were analyzed, of which 5,632 were preterm births. Prematurity increased from 7.9% in 2000 to 11.2% in 2013 –an average increase of 0.54% per year (r^2^ = 0.93)–with a growing share of moderate preterm births (32 to <37 weeks), which rose from 7.0% in 2000 to 9.7% in 2013. Between 2011 and 2013, multiple pregnancy (OR = 16.64; CI = 13.24–20.92), inadequate number of prenatal visits (OR = 2.81; CI = 2.51–3.15), Apgar score below 7 at 1 (OR = 4.07; CI = 3.55–4.67) and 5 minutes (OR = 10.88; CI = 7.71–15.36), low birth weight (OR = 38.75; CI = 33.72–44.55) and congenital malformations (OR = 3.18; CI = 2.14–4.74) were associated with preterm birth. A growing trend was observed for multiple pregnancies, with an average annual increase of 0.32% (r^2^ = 0.90), as well as for C-section birth (2.38% yearly increase). Of all newborn characteristics, Apgar score below 7 at 5 minutes (-0.19% per year) and low birth weight (-1.43%) decreased, whereas congenital malformations rose (0.20% per year).

**Conclusions:**

Efforts are required to prevent premature delivery, particularly during the moderate period, as well as greater care during the prenatal period towards expectant mothers bearing multiple pregnancies, birth defects, in addition to reducing C-section birth as it may be linked to preterm birth.

## Background

The prevalence of preterm births is high, with a tendency for further increase in several countries. In the United States, prematurity rose from 9.5% in 1981 to 12.3% in 2008, leveling off between 12% and 13% [1], while in Australia the rate of preterm births went from 6.8% in 1991 to 8.2% in 2009 [2].

In Brazil, a similar increase has been observed in preterm births, from 5% in 2005 to 11.8% in 2012; the state of Paraná has shown similar rates as the rest of the country, increasing from 6.2% to 11.9% in the same period [3]. For the municipality of Maringá, located in the northwest part of the state of Paraná, Brazil, the share of preterm births, which stood at 7.1% in 2005, increased to 13.2% in 2012 –higher than the rates for Brazil and Paraná State over the same period [3].

Preterm birth, defined as a birth that occurs prior to the 37^th^ week of gestation, is a serious public health problem, as it is linked to high rates of neonatal and child morbidity and mortality, as well as the possibility of disabilities during childhood and adult life [4–5]. It is estimated that about 15 million preterm births occur worldwide every year, and that over one million children die annually due to complications of prematurity [6]. Children born premature show high risks of handicaps such as neurological and cognitive development disabilities, disorders of the respiratory, liver, kidney, blood and sensory systems, nutritional and growth difficulties, as well as family stress and high public costs [5,7].

The etiology of preterm birth is complex and involves socioeconomic factors, the mother’s reproductive history, quality of prenatal care, maternal incidents during gestation, fetal traits, among others [8]. Possible etiologies of preterm birth include advanced maternal age, the growing use of assisted reproductive technologies, increase in multiple pregnancies and obstetric interventions [1,7].

Despite the consensus regarding the multifactor etiology of preterm birth, accrued knowledge is still insufficient to explain the rise in prematurity observed over the last few decades. Without deepening this knowledge, births prior to the 37^th^ gestational week may continue [6,9].

Research studies are needed to identify the risk factors related to prematurity [10–11], in order to plan interventions capable of reducing the occurrence of preterm births, considering they may vary according to the reality of each location.

The objective of this study was to analyze the trends in preterm births and associated factors in the city of Maringá, Paraná, Brazil. The expectation is to contribute to planning more effective interventions in order to reduce preterm births as well as perinatal and child morbidity and mortality.

## Materials and Methods

This was an ecological, time series and cross-section study of births listed in the Live Birth Information System (Sinasc) for residents of the municipality of Maringá, Paraná, in the period between 2000 and 2013.

Maringá, the third largest city in Paraná State with an estimated population of 391,753 in 2013, population density of 793 inhabitants per km², is the seat of the Maringá Metro Area (RMM) and the 15^th^ State Regional Health Office. It features a 98.6% rate of urbanization and Human Development Index (HDI) of 0.80, ranking sixth in the state and 67^th^ among all Brazilian cities [12].

To analyze the evolution of preterm births, data from 2000 to 2012 were obtained from Sinasc, and 2013 data were provided by the 15^th^ State Regional Health Office, because they were not yet available online on Datasus. The period between 2000 and 2013 was defined after exploring the thoroughness of the variables listed on Sinasc: gestational age, sex, Apgar at 1 and 5 minutes, race/color, birth weight, birth defect, mother’s age, marital status, mother’s educational level, profession, number of children born alive and stillborn, pregnancy type, type of delivery, number of prenatal visits and delivery site.

To determine the quality of the database and its potential use, the study analyzed the rate of undeclared variables—variables that were ignored or left blank. To that end, a scale was adapted to fit the reality of the satisfactory quality of information included into Sinasc in the state of Paraná: *excellent*, a percentage of undeclared variables below 1%; *good*, between 1% and 2.99%; *regular*, from 3% to 6.99%; and *poor*, when the percentage of undeclared variables is equal or greater than 7% [13].

Overall, the year with the worst rate of data completion was 1999, in contrast with 2013 when all variables were regarded as having excellent rates of completion. Accordingly, it was decided not to include the year 1999.

Preterm birth rates were calculated according to gestational age (GA): under 28 weeks (extreme prematurity); from 28 to <32 weeks (very premature); from 32 to <37 weeks (moderate preterm birth); and <37 weeks (WHO, 2012). Also analyzed were the delivery site (hospital or others), the mother’s age (<20; from 20 to 34 or ≥ 35 years), partner (yes or no), education level (<8 or ≥ 8 years of schooling), parity (primigravida or multigravida), pregnancy type (single or multiple), type of delivery (vaginal or C-section), number of prenatal visits (<7 or ≥ 7 visits), sex of the newborn (female or male), Apgar at 1 and 5 minutes (<7 or ≥7), birth weight (<2,500 or ≥2,500 gr) and congenital malformations (yes or no).

Rates were calculated year-to-year for trend analysis of preterm birth and trends in the characteristics of the mother, gestation and delivery, and newborn. To analyze the factors associated with preterm birth, the data were clustered into two three-year periods (2000 to 2002 and 2011 to 2013), using odds ratio (OR), with a confidence interval (CI) of 95%.

The polynomial regression model was used for trend analysis, in which preterm birth rates were regarded as dependent variables (y) and the years of the study were the dependent variable (x). The ‘year’ variable was transformed into the year-centered variable (x-2006) and the series were smoothed using a three-point moving average. The linear (y = β_0_+β_1_x_1_), quadratic (y = β_0_+β_1_x_1_+β_2_x_2_), and cubic polynomial regression models (y = β_0_+β_1_x_1_+β_2_x_2_+β_3_x_3_) were tested. Any trend whose estimated model reached a *p-value* <0.05 was considered significant. In order to select the best model, the choice for best model also took into consideration the analysis of the scatter plot, the value of the coefficient of determination (r^2^) and residual analysis (assumption of real homoscedasticity). When all criteria were significant for more than one model and the coefficient of determination was similar, the simpler model was chosen. All analyses were carried out using SPSS software, version 20.1.

The research project was approved by the Standing Committee for Ethics in Research of the Paraná State Health Secretariat/Workers Hospital (decision 406,927/2013). All data were obtained from public databases (http://datasus.saude.gov.br/). All data were anonymized.

## Results

**Fig 1:**
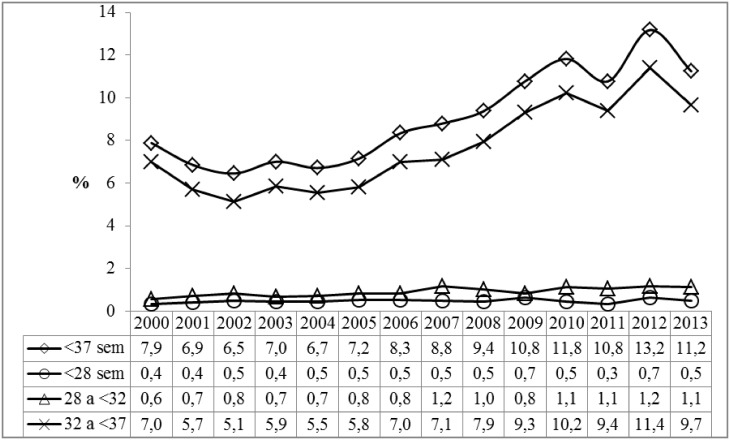
Rate of preterm births in relation to total births, according to gestational age, 2000 to 2013. Maringá, PR, Brazil.

The study analyzed 61,634 live births by mothers residing in Maringá, in the period between 2000 and 2013, of which 5,632 (9.1%) were preterm births. In 2000, the rate of preterm births was 7.9%, rising to 11.2% in 2013, with a larger share of moderate preterm births (from 32 to <37 weeks), accounting for 84.8% of total preterm births analyzed, and which went from 7.0% in 2000 to 9.7% in 2013 (Fig 1), with a relative increase of 38.6% in that period.

Trend analysis evidenced an average annual increase in preterm births of 0.54% (r^2^ = 0.93), when calculated in relation to all births in each year. Moderate preterm births—between 32 and <37 weeks—were the greatest contributors to the growth in prematurity, with an average increase of 0.49% per year (r^2^ = 0.91). Behavioral analysis of the different stages of prematurity—calculating the percentages in relation to all preterm births—showed a negative trend for extreme premature births (-0.43% per year) and very premature births (-0.15%), and a growing trend for moderately premature births (0.73% per year) (Table 1).

**Table 1:** Trend models of the rate of preterm births, according to gestational age stages. Maringá-PR, Brazil, 2000 to 2013.

**IG** ^a^	**Model**	**R^2^**	***p***	**Trend**
<37 wks	y = 8.64 + 0.54x	0.93	<0.001	Growth
<28 wks	y = 0.50 + 0.006x - 0.02x^2^	0.74	0.001	Growth
28 to <32	y = 0.89 + 0.04x	0.93	<0.001	Growth
32 to <37	y = 7.26 + 0.49x	0.91	<0.001	Growth
**IG** ^b^	**Model**	**R^2^**	***p***	**Trend**
<28 wks	y = 6.26—0.43x - 0.04x^2^ + 0.01x^3^	0.98	<0.001	Decrease
28 to <32	y = 10.48–0.15x	0.36	0.037	Decrease
32 to <37	y = 82.67 + 0.73x + 0.10x^2^–0.02x^3^	0.92	<0.001	Growth

^**a**^ Percentage of each prematurity component calculated in relation to total births in each year.

^**b**^ Percentage of each prematurity component calculated in relation to total preterm births in each year.

In the three-year period from 2000 to 2002, the following factors were associated with preterm birth: mother’s age under 20 years (OR = 1.34; *p* = 0.001) or 35 years or older (OR = 1.31; *p* = 0.012), having less than eight years of formal education (OR = 1.28; *p* = 0.001), and having a first pregnancy (OR = 1.21; *p* = 0.008). With regard to characteristics of gestation and delivery: multiple pregnancy (OR = 8.56; *p*<0.001), and having had less than seven prenatal visits (OR = 2.76; *p*<0.001). With regard to newborn characteristics: having had Apgar score below 7 at 1 minute (OR = 5.78; *p*<0.001), and at 5 minutes (OR = 12.97; *p*<0.001), having birth weight lower than 2,500 gr (OR = 36.72; *p*<0.001), and the presence of congenital malformations (OR = 4.63; *p*<0.001). In that three-year period, having a C-section birth was a protective factor for preterm birth (OR = 0.77; *p*<0.001) (Table 2).

**Table 2:** Factors associated with preterm birth. Maringá-PR, Brazil.

	2000–2002			2011–2013		
	< 37			< 37		
Variables	%	OR	(CI 95%)	%	OR	(CI 95%)
**Mother**						
**Age**						
< 20	8.6	1.34	1.12–1.59	12.5	1.1	0.93–1.31
20–34	6.6			11.4		
≥ 35	8.5	1.31	1.06–1.62	12.9	1.15	0.10–1.32
**Partner**						
Yes	6.8			11.5		
No	7.6	1.13	0.98–1.30	12.1	1.06	0.96–1.17
**Education level**						
< 8	8.2	1.28	1.11–1.47	12.8	1.12	0.96–1.31
≥ 8	6.5			11.6		
**Parity**						
Primigravida	7.6	1.21	1.05–1.39	11.5	0.96	0.85–1.06
Multigravida	6.4			11.9		
**Gestation and delivery**						
**Pregnancy type**						
Single	6.4			10.4		
Multiple	36.9	8.56	6.67–10.99	65.9	16.64	13.24–20.92
**Type of delivery**						
C-section	6.6	0.77	0.66–0.89	11.5	0.90	0.80–1.09
Vaginal	8.4			12.6		
**Prenatal visits**						
< 7	13	2.76	2.40–3.17	23	2.81	2.51–3.15
≥ 7	5.2			9.6		
**Delivery site**						
Hospital	7.1			11.7		
Others	10.3	1.45	0.53–4.23	15.8	1.41	0.41–4.85
**Newborn**						
**Sex**						
Female	7.3			11.6		
Male	6.9	0.94	0.82–1.08	11.9	1.03	0.93–1.13
**Apgar 1**						
< 7	26.2	5.78	4.84–6.90	31.3	4.07	3.55–4.67
≥ 7	5.8			10.1		
**Apgar 5**						
< 7	47.6	12.97	9.33–8.02	58.1	10.88	7.71–15.36
≥ 7	6.6			11.3		
**Birth weight**						
< 2500	55.4	36.72	31.16–43.28	70.6	38.76	33.72–44.55
≥ 2500	3.3			5.8		
**Malformation**						
Yes	25.6	4.63	3.06–7.01	29.4	3.18	2.14–4.74
No	6.9			11.6		

For the second three-year period (2011 to 2013), only the variables regarding gestation and delivery and the newborn showed a significant association with preterm birth. Multiple pregnancy (OR = 16.64; *p*<0.001), having had fewer than seven prenatal visits (OR = 2.81; *p*<0.001); Apgar score below 7 at 1 (OR = 4.07; *p*<0.001) and 5 minutes (OR = 10.88; *p*<0.001), as well as low birth weight (OR = 38.75; *p*<0.001) and congenital malformations (OR = 3.18; *p*<0.001), were factors associated with preterm birth (Table 2).

The result of the trend analysis of the factors associated with preterm birth showed a reduction in the number of births of children from teenage mothers (-0.97% per year), and an increase in preterm births among women without a partner (0.85% per year) and with eight or more years of formal schooling (mean percentage of 83.26% and a 1.5% increase per year). With regard to pregnancy type, a growing trend was observed in multiple pregnancy births, with an annual increase of 0.32%, and high explanatory power for the model (r^2^ = 0.90). C-section birth showed a tendency towards increase, only among premature births, with an average annual increase of 2.38% (Table 3). It is noteworthy that C-section birth was carried out in 79.7% of all deliveries during that period.

**Table 3:** Trend models for the rate of preterm births, according to variables of mother, gestation and delivery, and newborn. Maringá-PR, Brazil, 2000 to 2013.

Variables	Model	R^2^	*p*	Trend^a^
**Mother**				
**Age**				
< 20	y = 16.17–0.97x	0.81	<0.001	↓
20–34	y = 69.84 + 0.71x	0.76	<0.001	↑
≥ 35	y = 13.82 + 0.16x	0.29	0.068	-
**Partner**				
Yes	y = 57.68–0.64x	0.36	0.038	↓
No	y = 44.40 + 0.85x—0.19x^2^	0.59	0.007	↑
**Education level**				
< 8	y = 16.77–1.50x + 0.26x^2–^0.02x^3^	0.99	<0.001	↓
≥ 8	y = 83.26 + 1.50x—0.28x^2^ + 0.02x^3^	0.99	<0.001	↑
**Parity**				
Primigravida	y = 51.49–0.25x	0.09	0.353	-
Multigravida	y = 48.50 + 0.25x	0.09	0.353	-
**Gestation and delivery**				
**Pregnancy type**				
Single	y = 83.86–0.32x + 0.11x^2^	0.90	<0.001	↓
Multiple	y = 16.14 + 0.32x—0.11x^2^	0.90	<0.001	↑
**Type of delivery**				
C-section	y = 67.99 + 2.38x + 0.24x^2–^0.05x^3^	0.90	<0.001	↑
Vaginal	y = 32.01–2.38x—0.24x^2^ + 0.05x^3^	0.90	<0.001	↓
**Prenatal visits**				
< 7	y = 38.41–1.12x	0.96	<0.001	↓
≥ 7	y = 61.31 + 0.12x	0.96	<0.001	↑
**Delivery site**				
Hospital	y = 99.68 + 0.02x	0.21	0.136	-
Others	y = 0.32–0.02x	0.21	0.136	-
**Newborn**				
**Sex**				
Female	y = 48.47–0.18x	0.36	0.039	↓
Male	y = 51.50 + 0.18x	0.33	0.049	↑
**Apgar 1**				
< 7	y = 23.25–0.26x	0.24	0.103	-
≥ 7	y = 76.20 + 0.38x	0.39	0.029	↑
**Apgar 5**				
< 7	y = 5.92–0.19x	0.49	0.011	↓
≥ 7	y = 93.58 + 0.31x	0.69	0.001	↑
**Birth weight**				
< 2500	y = 65.69–1.43x—0.38x^2^ + 0.05x^3^	0.85	<0.001	↓
≥ 2500	y = 34.30 + 1.44x + 0.37x^2–^0.05x^3^	0.86	<0.001	↑
**Malformation**				
Yes	y = 1.89 + 0.20x + 0.04x^2–^0.01x^3^	0.77	0.002	↓/↑
No	y = 98.18 + 0.53x—0.13x^2^	0.62	0.005	↑

^**a**^ ↑ Growth; ↓ Decrease;—Constant; ↑/↓ Growth/Decrease; ↓/↑ Decrease/Growth.

Percentages component calculated in relation to total births in each year.

Table 3 further shows a decreasing trend of preterm births for children with Apgar score below 7 at 5 minutes (-0.19% per year) and low birth weight (-1.43% per year). Premature births of children with congenital malformations showed an increasing trend (0.20% per year).

## Discussion

This study presents data consistent with those found in the literature, with a significant increase in preterm birth [14], which occurred especially in the moderate period. Extreme prematurity and very premature births showed a decreasing trend when analyzed in relation to all preterm births.

In Brazil, research and investments have focused on newborns with fewer than 32 weeks of gestation. However, the same has not occurred with moderately preterm babies, especially borderline ones (34 to <37 weeks), which are largely ignored and left without early detection of problems related to their development [15].

With regard to the factors associated with preterm birth, it is possible to detect a transition in the epidemiological profile of maternal factors, signaled by the changes seen in the results found in the analysis of maternal sociodemographic variables, which showed no link to preterm birth during the three-year period from 2011 to 2013. Similar results were observed in a case-control study on preterm births carried out in the city of Londrina, state of Paraná, in which the mother’s age, education level and parity were not associated with prematurity, either [16].

Both in Brazil and others middle income countries, the increase of preterm births has been recorded in a context of demographic and epidemiological transition, with modifications in some characteristics of the population of women including a diminution of fecundity rate, and decrease amount of marriage [17]. During this process it has also been observed an increase of women education level and delayed maternal age at first child [17,18]. These changes may aid in the comprehension of the challenges presented by the context of preterm birth.

With regard to gestation and delivery variables, only multiple pregnancy and the insufficient number of prenatal visits remained associated with prematurity in both three-year periods.

Research studies indicate that perinatal morbidity/mortality and the occurrence of prematurity are higher in multiple pregnancies, as a result of distention of uterine fibers, causing early maturing, as well as other common complications in pregnancies with more than one conceived fetus [7], and that a significant share of preterm births can be regarded as avoidable with adequate assistance to the mother during prenatal visits. Quality prenatal care goes beyond the number of appointments and routine assistance in prenatal exams, requiring more than access to health services and exams, in that professionals responsible for care have and utilize clinical and scientific knowledge to provide the necessary support to intervene in adverse situations, such as incidents during gestation and multiple pregnancies, thus contributing to reduce preterm births [19].

The results of this study showed that an insufficient number of prenatal visits was significantly associated with preterm birth (OR = 2.76 and OR = 2.81, for 2000–2002 and 2011–2013, respectively). It should be taken into account that if a birth occurs prematurely, a lower number of visits may have occurred because there was not enough time for them to take place, particularly because appointments are scheduled with greater frequency starting in the 28^th^ week of gestation. However, it is important to consider that 84.8% of premature births analyzed in this study were moderately premature births (32 to <37 weeks), and that some characteristics such as multiple pregnancy and gestations with fetal malformations—often linked to preterm births—indicate the need for a closer look by health services towards expectant mothers with these characteristics, by scheduling more prenatal visits, given that six is only the minimum recommended number of appointments [20].

C-section birth was performed in 79.7% of all deliveries in that period—much higher than the upper limit of 15% recommended by the World Health Organization [21]. Despite being considered a protective factor for preterm birth in the first three-year period, it came very close to becoming a risk in the last three-year period, and showed a growing trend among premature births, likely indicating a change in the future if maternal and healthcare conditions remain the same. It should be taken into account that a large share of deliveries in moderately premature children happen due to elective C-sections, with no clear purpose for their early performance [15]. New studies are suggested on factors associated with preterm birth, according to gestational age, also considering the reason for the referral for C-section birth.

Polynomial regression analysis showed that preterm birth has increased, and although it is possible to observe that factors related to prematurity are mostly decreasing, the sociodemographic improvement observed was not enough to reverse the scenario of prematurity in Maringá.

In several developed countries, there are references to this same growing trend in prematurity, attributed in large part to obstetric interventions, such as elective C-section, assisted reproduction and increase in multiple births [1,7]. One study carried out using secondary data in the United States suggests that the increase in premature births is more related to changes in obstetric practice than to changes in maternal variables. The results of that study indicate that even if socioeconomic variables remained unchanged over the years, prematurity rates would be very similar to current ones, due to the marked increase in obstetric interventions [22]. The same may be happening in Maringá, given that prematurity increased despite improved sociodemographic conditions.

The data presented herein show a growing trend in multiple pregnancies among women who had premature births, possibly due to earlier motherhood and the popularization of assisted fertilization procedures, particularly those featuring induced ovulation [7]. Children born after the use of assisted reproductive technology have significantly higher rates of prematurity. Moreover, gestations resulting from reproductive assistance carry a high risk of gestational morbidities, as well as other adverse reproductive results, such as congenital malformations and neonatal mortality. It is also important to consider the need for employing proper techniques to control multiple gestations whenever assisted reproduction technologies are utilized [23].

Also noteworthy is the increase in pregnancies among women 35 or older (8.5% and 12.9%, in the first and second three-year periods, respectively), a factor that may contribute to the increase in gestations with birth defects, considering that these pregnancies are often subjected to ovular stimulation, artificial insemination, in addition to frequently showing high blood pressure, diabetes and choice for elective C-section—all of which are risk factors for preterm birth.

In that regard, pre-conception interventions, such as iron supplementation to prevent neural tube closing defects [24] and gestational monitoring, stand out as some of the factors that make it possible to reduce the likelihood of birth defects, given that morphological development of the fetus and most maternal-fetal morbidities can be prevented, diagnosed and treated early, allowing for intrauterine therapy in cases of minor malformations and interventions to the mother’s health to prevent adverse events such as preterm birth and mother/child mortality [25].

From the polynomial regression model, it was possible to detect that favorable vitality conditions at birth (Apgar greater than or equal to 7 at 1 and 5 minutes, and adequate birth weight) have increased among premature births, which may be linked to improved preparation in delivery care, as well as the marked increase in moderately premature newborns.

It is also necessary to consider the complexity that involves the occurrence of preterm births, in that prematurity may be associated with several other factors such as stress during gestation and maternal incidents, which were not analyzed in this study because they were not available in Sinasc; they require more profound population studies, including analysis of births according to prematurity stages at the local level.

Knowing and understanding the complex process of preterm birth and its interfering factors is essential to provide quality assistance to the mother and child, as well as to perfect and rationalize the care given during all stages or the reproductive cycle, in order to prioritize actions of prevention, recovery and life maintenance, and furthermore, to direct and adopt preventive and curative measures in consonance with local reality [8].

Some limitations of this study are noteworthy, such as those related to information obtained from secondary databases, subject to data completion and reliability. Another limitation concerns the impossibility of working with borderline prematurity, from 34 to <37 weeks, given the manner of data collection regarding gestational age by Sinasc—until 2009, it was categorized directly into intervals; only starting in 2010 did it come to be recorded as number of weeks of gestation [26].

## Conclusions

The present study evidenced a growing trend for preterm birth in a mid-sized city in southern Brazil, which occurred mainly in the moderate period. It is important to maintain health actions and programs that are showing a positive effect on very premature and extremely premature births in Brazil, but health actions and programs geared towards moderate preterm birth are necessary as well, as they concentrate the highest number of premature births.

To that end, according to the results of this study, closer attention must be paid during the prenatal period to expectant mothers with multiple pregnancy and fetuses with birth defects, which have shown to be a risk for this unfavorable event.

Also important is the need for further and more detailed population studies, including an analysis of preterm births and associated factors according to gestational age.
